# Autoimmune Vasculitis Causing Acute Bilateral Lower Limb Paralysis

**DOI:** 10.7759/cureus.27651

**Published:** 2022-08-03

**Authors:** Ayuko Tokonami, Ryuichi Ohta, Noritaka Katagiri, Naho Yoshioka, Fumiko Yamane, Chiaki Sano

**Affiliations:** 1 Medicine, Shimane University Faculty of Medicine, Izumo, JPN; 2 Community Care, Unnan City Hospital, Unnan, JPN; 3 Family Medicine, Shimane University Faculty of Medicine, Izumo, JPN; 4 Community Medicine Management, Shimane University Faculty of Medicine, Izumo, JPN

**Keywords:** japan, rural, older patient, eosinophilic granulomatosis with polyangiitis, granulomatosis with polyangiitis, microscopic polyangiitis, polyarteritis nodosa, paralysis, autoimmune vasculitis

## Abstract

Autoimmune vasculitis is an autoimmune disease that causes various systemic symptoms, such as fever, fatigue, joint pain, and night sweats. Its clinical course depends on the severity of the inflammation, which can cause acute clinical progression of symptoms. Moreover, when the inflammation of the arteries occurs in the deeper parts of the body, a biopsy may be difficult to perform. Here, we report a case of autoimmune vasculitis in an elderly man who visited our hospital with a chief complaint of muscle pain and fever triggered by a rapid paralysis of both lower limbs. Autoimmune vasculitis can cause a variety of systemic symptoms depending on the size of involved arteries, and its clinical course depends on the severity of the inflammation. Prompt diagnosis and simultaneous treatment of symptoms, excluding other likely diseases, prevent the development of severe and long-term complications of autoimmune vasculitis.

## Introduction

Autoimmune vasculitis is an autoimmune disease that causes various systemic symptoms, such as fever, fatigue, joint pain, and night sweats [1,2]. There are several types of autoimmune vasculitis based on the size of involved arteries [1]. Inflammation of the small or middle arteries can cause various systemic symptoms, such as nephritis, hepatitis, neuropathy, and interstitial pneumonia [3]. The four major autoimmune vasculitis types are microscopic polyangiitis, granulomatosis with polyangiitis (GPA), eosinophilic GPA, and polyarteritis nodosa [4]. Vasculitis can be diagnosed based on symptoms and pathological findings of necrotic or granulomatous inflammation on the arterial walls vis biopsy [2,3]. Treatment with steroids and immunosuppressive drugs can prevent the development of severe complications of vasculitis [5].

The clinical course of autoimmune vasculitis depends on the severity of the inflammation, which can cause acute clinical progression of symptoms [6]. Ideally, pathological examinations are performed to diagnose vasculitis; however, autoimmune vasculitis with a very acute clinical course cannot be diagnosed by such examinations [7]. Moreover, when the inflammation of the arteries occurs in the deeper parts of the body, biopsy itself can be challenging [3]. Here, we report a case of autoimmune vasculitis in an elderly man with a chief complaint of muscle pain and fever. Acute bilateral lower limb paralysis was observed during the clinical course. He was diagnosed with small-to-medium-sized vasculitis based on the clinical symptoms and inflammatory changes in the arterial walls on computed tomography with contrast and laboratory tests. The patient was eventually diagnosed with GPA based on the serological results. He was successfully treated with intravenous prednisolone pulse, cyclophosphamide, and rituximab and partially recovered from lower leg paralysis. This case highlights the importance of promptly diagnosing autoimmune vasculitis by excluding various critical diseases and providing related treatments to prevent developing severe and long-term complications of vasculitis.

## Case presentation

An 82-year-old man who can independently perform activities of daily living (ADL) presented to our hospital with a chief complaint of fever, fatigue, and muscle pain while walking. He was admitted to the hospital for further investigation of the fever with a high inflammatory response. He had a history of prostate cancer at age of 60 years, treated with radical prostatectomy and other comorbidities, including bronchial asthma, Barrett's esophagus, hyperlipidemia, and chronic obstructive pulmonary disease. He has been a smoker until the age of 60 years. His medications include pravastatin sodium (10 mg), budesonide formoterol fumarate hydrate, and tipepidine hibenzate. 

His vital signs on admission were as follows: temperature was 36.8 °C, blood pressure was 117/62 mmHg, pulse rate was 95 beats/min, respiratory rate was 16 times/min, and SpO_2_ was 98% (room air). Physical examination revealed tenderness in the deep lower right abdomen and right dominant lateral abdomen. A manual muscle test (MMT) results were as follows: iliopsoas muscle, 4+/5 -; quadriceps muscle, 5/5; biceps femoris muscle, 5/5; triceps femoris muscle, 5/5; and tibialis anterior muscle, 5/5. During the examination, the patient experienced pain induced by hip flexion. After admission, the patient developed a fever (temperature > 39°C) and tachypnea. The laboratory showed high inflammatory condition (Table 1).

**Table 1:** Initial laboratory data of the patient. eGFR: estimated glomerular filtration rate; CK: creatine kinase; TSH: thyroid-stimulating hormone; Ig: immunoglobulin; HCV: hepatitis C virus; SARS-CoV-2: severe acute respiratory syndrome coronavirus 2; HBs: hepatitis B surface antigen; HBc: hepatitis B core antigen

Marker	Level	Reference
White blood cells	4.00	3.5-9.1 × 10^3^/μL
Neutrophils	77.9	44.0-72.0%
Lymphocytes	10.2	18.0-59.0%
Monocytes	10.7	0.0-12.0%
Eosinophils	0.8	0.0-10.0%
Basophils	0.4	0.0-3.0%
Red blood cells	3.98	3.76-5.50 × 10^6^/μL
Hemoglobin	11.7	11.3-15.2 g/dL
Hematocrit	36.5	33.4-44.9%
Mean corpuscular volume	91.5	79.0-100.0 fL
Platelets	26.5	13.0-36.9 × 10^4^/μL
Erythrocyte sedimentation rate	89	2-10 mm/h
Total protein	5.9	6.5-8.3 g/dL
Albumin	3.0	3.8-5.3 g/dL
Total bilirubin	0.2	0.2-1.2 mg/dL
Aspartate aminotransferase	41	8-38 IU/L
Alanine aminotransferase	25	4-43 IU/L
Alkaline phosphatase	118	106-322 U/L
γ-Glutamyl transpeptidase	72	<48 IU/L
Lactate dehydrogenase	366	121-245 U/L
Blood urea nitrogen	11.3	8-20 mg/dL
Creatinine	0.66	0.40-1.10 mg/dL
eGFR	86.2	>60.0 mL/min/L
Serum Na	137	135-150 mEq/L
Serum K	4.1	3.5-5.3 mEq/L
Serum Cl	101	98-110 mEq/L
Ferritin	391.7	14.4-303.7 ng/mL
CK	27	56-244 U/L
C-reactive protein	13.18	<0.30 mg/dL
TSH	0.35	0.35-4.94 μIU/mL
Free T4	0.9	0.70-1.48 ng/dL
IgG	1069	870-1700 mg/dL
IgM	19	35-220 mg/dL
IgA	188	110-410 mg/dL
HBs antigen	0.0	IU/mL
HBs antibody	0.0	mIU/mL
HBc antibody	0.0	S/CO
HCV antibody	0.0	S/CO
Syphilis treponema antibody	0.0	S/CO
SARS-CoV-2 antigen	Negative	-
Urine test		
Leukocyte	Negative	-
Nitrite	Negative	-
Protein	Negative	-
Glucose	Negative	-
Urobilinogen	Negative	-
Bilirubin	Negative	-
Ketone	Negative	-
Blood	Negative	-
pH	6.0	-
Specific gravity	1.024	-

We considered the possibility of bacterial translocation from the gastrointestinal tract based on shaking chill and persistent fever and initiated intravenous cefmetazole treatment. The urine and blood culture results were negative. 

**Figure 1:**
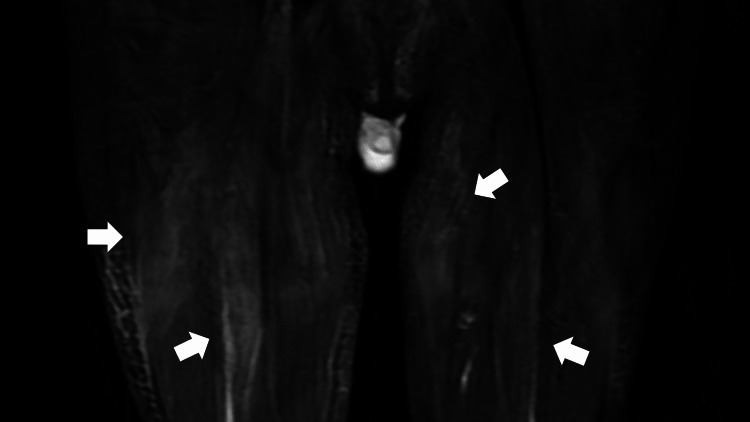
A femoral magnetic resonance imaging scan (coronal plane) showing the bilateral hip and thigh muscles with a high signal (arrows).

His fever continued (38°C) along with muscle pain; etodolac tablets (200 mg) were started to mitigate these. Additional tests were negative for antinuclear and antiphospholipid antibodies and revealed mildly elevated aldolase levels. Short tau inversion recovery magnetic resonance imaging (MRI) of the thighs revealed high signals in the bilateral gluteal and thigh musculatures (Figure 1).

A biopsy of the right semitendinosus muscle was performed, which showed negative findings for vasculitis and hematological malignancy. He was suspected of having polymyalgia rheumatica and was treated with 20 mg of prednisolone, which was increased to 30 mg two days later owing to persistent fever and exacerbated systemic muscle pain.

**Figure 2:**
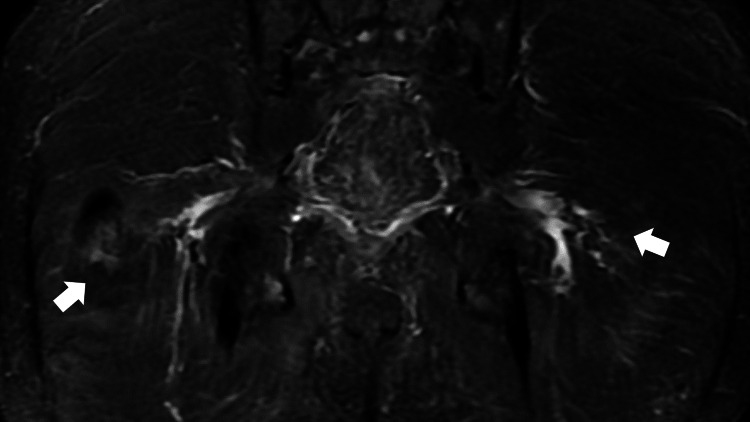
A pelvic magnetic resonance imaging scan (coronal plane) showing high signals in the bilateral sciatic nerves (arrows)

On day 18 of hospitalization, rapidly progressive paralysis of both the lower limbs appeared. Neurological examination revealed decreased tendon reflexes and a loss of rectoanal reflexes, and sensory loss in the lesions of L4 to S2. Cerebrospinal fluid analysis revealed protein-cell dissociation in the cerebrospinal fluid (protein, 117 mg/dL; cell counts: 21/μL). Lumbar and pelvic magnetic resonance imaging (MRI) showed high signals in the bilateral sciatic nerves without no compression in spinal cord space from Th8 to L5 to induce paralysis (Figure 2).

**Figure 3:**
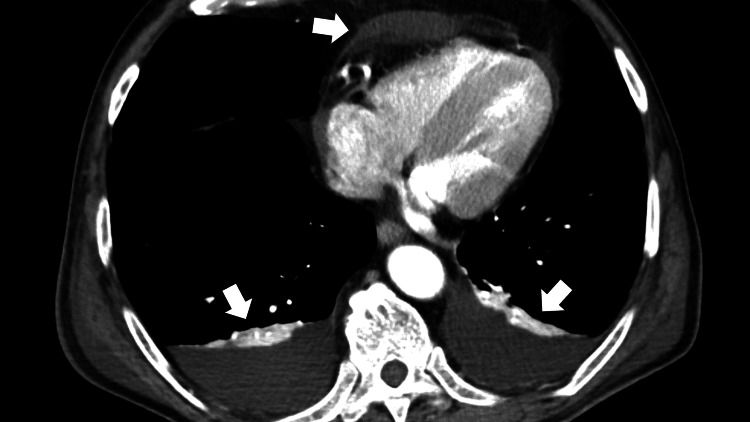
An enhanced abdominal computed tomography scan (transverse plane) showing generalized edema of the periarterial lesions around the superior and inferior mesenteric arteries (arrows).

**Figure 4:**
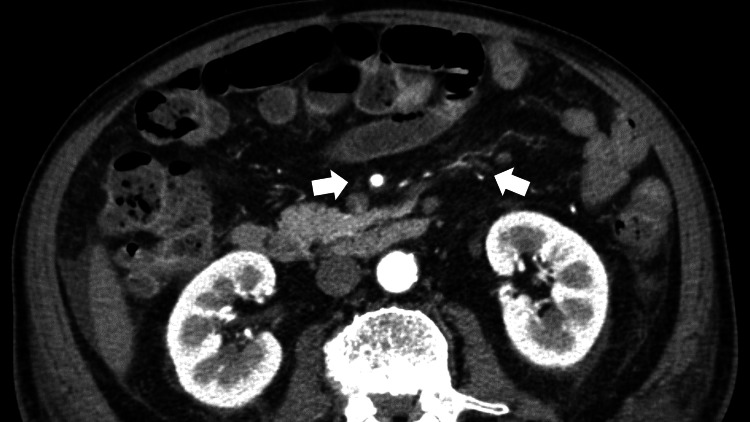
An enhanced abdominal computed tomography scan (transverse plane) showing effusions of pericardial and pleural fluid (white arrows)

His additional clinical history clarified proceeding epistaxis, cough, stomatitis, dyspnea, systemic edema, and hypertension. Repeat antibody tests were negative for perinuclear antineutrophil cytoplasmic antibodies (ANCA), but the cytoplasmic ANCA levels (C-ANCA) were mildly elevated (2.7 U/mL). Lumbar MRI did not reveal an obvious cauda equina syndrome. Computed tomography (CT) of the thoracic and pelvic regions showed generalized edema of the periarterial lesions around the superior and inferior mesenteric arteries (Figure 3) and effusions of pericardial and pleural fluids (Figure 4). There was no finding of the thrombosis of the artery of Adamkiewicz in the CT. 

Based on the clinical course, the patient was presumed to have an acute progressive inflammatory condition caused by GPA. On day 19 of hospitalization, 1000 mg of methylprednisolone was administered for three days. Owing to the presence of protein-cell dissociation of the spinal fluid and acute progressive paralysis of the lower limbs, a diagnosis of Guillain-Barré syndrome was made; 4 mg/kg of intravenous immunoglobulin was administered for five days for the acute demyelinating condition. Ceftriaxone of 4 g per day and acyclovir of 1500 mg per day were administered to treat possible viral/bacterial meningitis. No obvious malignant findings were identified on muscle biopsy, spinal fluid analysis, or peripheral blood pathology; however, the patient’s symptoms progressed. Thus, 500 mg of cyclophosphamide pulse 500 was added for possible exacerbation of granulomatosis with polyangiitis. He also had urinary retention, was suspected of a neurogenic bladder, and was treated with a urinary catheter.

On day 29 of hospitalization, because of an inadequate response to cyclophosphamide pulse therapy, rituximab 500 mg was started. This caused marked relief of the abdominal pain. Moreover, the progression of muscle weakness in both lower limbs stopped, and symptoms in both lower limbs gradually improved with rehabilitation. The patient was treated with four weekly infusions of rituximab. The patient's paralysis was alleviated, and we removed his urinary catheter on the 41st day. He was transferred to the rehabilitation ward before being discharged home.

## Discussion

In the present case, the patient was diagnosed with GPA based on the clinical course and imaging findings, demonstrating rapidly progressing muscle weakness in the lower extremities. The prompt administration of steroid pulse, cyclophosphamide, and rituximab suppressed the progression of paralysis in the lower extremities. This case illustrates that vasculitis can develop rapidly, requiring prompt treatment based on clinical judgment, and that treatment delay of rapidly progressing vasculitis with neurological symptoms can lead to long-term complications. Treatment delays with such an aggressive form of vasculitis with rapidly progressing neurological symptoms may also lead to long-term complications requiring intensive treatment. Our patient's paralysis due to rapidly progressive polyangiitis of both the lower extremities progressed from an unknown fever. Contrast-enhanced CT and MRI of the abdominal and pelvic regions revealed edema in the small- and medium-sized blood vessels and abnormal neuromuscular signals, leading to a clinical diagnosis of small- and medium-sized vasculitis [3,8]. Additionally, the patient received steroid pulse and immunosuppressive therapies with cyclophosphamide. Vasculitis is a chronic progressive condition, and biopsies of the purpura and peripheral nerves are usually performed to confirm the disease based on pathological findings [9]. However, when the symptoms progress rapidly, as in the present case, treatment should be initiated immediately based on the clinical diagnosis [10]. Previous studies have shown that the longer the time between the onset of neuroinflammation and treatment, the more severe the decline in the patient's ability to perform ADLs [11,12]. Early diagnosis and immunosuppressive treatment are important for the successful treatment of vasculitis [11,12]. This progression may determine the disease prognosis in community hospitals with many elderly patients [13]. General practitioners should have a broad scope of practice to identify and treat vasculitis.

It is also difficult to accurately diagnose vasculitis with rapidly progressing neurological symptoms. The sensitivity of histological tests, which are important for diagnosing vasculitis, may not be high, and multiple biopsies are often needed [9]. However, in the case of rapidly progressing vasculitis, long-term complications may occur due to a delay in treatment initiation; therefore, early intervention is important [6,7]. In the present case, skin and muscle biopsies failed to indicate a clear pathology. A diagnosis of vasculitis was made based on the imaging findings and clinical course, and the patient was treated immediately. As a result, the neuropathy progression was minimized as much as possible. The diagnosis of microscopic polyangiitis, GPA, eosinophilic GPA, and polyarteritis nodosa is often made based on pathological findings [3,5]. As in this case, performing antibody tests is important for early diagnosis and treatment. For vasculitis, clinicians should be aware that the diagnostic process and treatment differ depending on the speed of the clinical course of the disease.

In a community hospital setting, prompt treatment determines the prognosis of patients with rapid paralysis progression due to vasculitis. Thus, it is necessary for clinicians to consider the possibility of various diseases and proceed with the examination and treatment simultaneously. In a rural context, analyzing and responding to the complaints of elderly patients is necessary. Owing to the aging population, an increasing number of patients are presenting at community hospitals with fever and indeterminate complaints [14]. In many cases, voluntary care often alerts patients and preserves their quality of life [15,16]. However, some patients do not promptly seek medical care and visit hospitals with gradually increasing and progressive pathologies, as in the present case [17,18]. To ensure the provision of effective community medical care services in the future, it is necessary to promote appropriate and prompt visits to healthcare facilities. Moreover, it is necessary to educate patients in the community on the symptoms of disease recurrence and appropriate treatment behaviors [19,20]. The ability to respond to rapidly progressive conditions based on appropriate treatment behaviors is important for facilitating the diagnosis of chronic inflammatory diseases, including vasculitis.

## Conclusions

We successfully treated a patient with rapidly progressive polyneuritis and paraplegia with steroid pulse, cyclophosphamide, and rituximab, although a diagnosis of GPA was not made based on biopsy results. It is important to initiate treatment for rapidly progressing vasculitis with neurological symptoms as early as possible. Elderly patients often present with undefined complaints, and general practitioners at community hospitals need to have a broad scope of practice to facilitate the diagnosis and treatment of such cases.
